# The impact of adverse events in the intensive care unit on hospital mortality and length of stay

**DOI:** 10.1186/1472-6963-8-259

**Published:** 2008-12-17

**Authors:** Alan J Forster, Kwadwo Kyeremanteng, Jon Hooper, Kaveh G Shojania, Carl van Walraven

**Affiliations:** 1 Department of Medicine, University of Ottawa, Ottawa, Canada; 2 Clinical Epidemiology Program, Ottawa Health Research Institute, Ottawa, Canada; 3 Department of Critical Care, University of Ottawa, Ottawa, Canada; 4 Department of Medicine, University of Toronto, Toronto, Canada; 5 Institute for Clinical Evaluative Sciences, Toronto, Canada

## Abstract

**Background:**

Adverse events (AEs) are patient injuries caused by medical care. Previous studies have reported increased mortality rates and prolonged hospital length of stay in patients having an AE. However, these studies have not adequately accounted for potential biases which might influence these associations. We performed this study to measure the independent influence of intensive care unit (ICU) based AEs on in-hospital mortality and hospital length of stay.

**Methods:**

Prospective cohort study in an academic tertiary-care ICU. Patients were monitored daily for adverse clinical occurrences. Data about adverse clinical occurrences were reviewed by a multidisciplinary team who rated whether they were AEs and whether they were preventable. We determined the association of AEs in the ICU with time to death and time to hospital discharge using multivariable survival analysis models.

**Results:**

We evaluated 207 critically ill patients (81% required mechanical ventilation, median Glasgow Coma Scale = 8, median predicted mortality = 31%). Observed mortality rate and hospital length of stay were 25% (95% CI 19%–31%) and 15 days (IQR 8–34 days), respectively. ICU-based AEs and preventable AEs occurred in 40 patients (19%, 95% CI 15%–25%) and 21 patients (10%, 95% CI 7%–15%), respectively. ICU-based AEs and preventable AEs were not significantly associated with time to in-hospital death (HR = 0.93, 95% CI 0.44–1.98 and HR = 0.72 95% CI 0.25–2.04, respectively). ICU-based AEs and preventable AEs were independently associated with time to hospital discharge ((HR = 0.50, 95% CI 0.31–0.81 and HR = 0.46 95% CI 0.23–0.91, respectively)). ICU-based AEs were associated with an average increase in hospital length of stay of 31 days.

**Conclusion:**

The impact of AEs on hospital length of stay was clinically relevant. Larger studies are needed to conclusively measure the association between preventable AEs and patient outcomes.

## Background

Adverse events (AEs) refer to patient injuries caused by medical care [1]. Widely quoted estimates of the mortality attributable to medical error (44,000 to 98-000 deaths per year in US hospitals [1]) are based on retrospective studies that utilize chart reviews [2,3]. These studies measured AE rates along with the proportion of AE patients who died. This methodology does not adequately assess the causal relationship between AEs and subsequent hospital outcomes [4].

There are two reasons why chart reviews could lead to flawed measures of how AEs impact patient outcomes. First, the reviewer who determines AE status knows the patient's outcome. This knowledge biases the assessment of whether an AE (or error) actually occurred, as well as the extent to which the AE contributed to the bad outcome [5]. Second, acutely ill patients have substantial risks of both poor outcomes and AEs. Thus, the association of AEs with poor outcomes may be due to confounding [4]. An example of this phenomenon is nicely illustrated in a recent secondary analysis of data from a randomized controlled trial showing that patients who received incorrect doses of a study drug were more likely to experience adverse clinical outcomes, irrespective of whether the treatment received was placebo or the active agent [6].

Determining the true burden attributable to AEs is important for two reasons. First, policy makers and clinicians require an accurate measure of the influence AEs have on patient outcomes to gauge their importance. Second, safety strategies need to target the most frequent and clinically important AEs for the most efficient use of resources. We therefore undertook a prospective study to accurately quantify the effect of all AEs and preventable AEs on hospital mortality and length of stay for a cohort of intensive care unit (ICU) patients. We studied this population because ICU patients have well developed risk adjustment instruments and a sufficiently high rate of AEs and death to facilitate identifying an association between AEs and outcomes.

## Methods

For three consecutive months in 2006, we prospectively followed all patients admitted to the ICU at the Ottawa Hospital's Civic Campus from ICU admission until hospital discharge or death. Patient observation was censored 60 days after discharge from the ICU. The Ottawa Hospital Civic Campus is a tertiary care academic hospital providing a range of clinical services to the people of Eastern Ontario, including a regional trauma service. The Ottawa Hospital Research Ethics Board approved the protocol.

### Baseline Data Collection

We collected baseline information on each patient when they were admitted to the ICU and 24 hours later. This information included basic demographic data, indication for ICU admission, clinical information (including the patient's baseline chronic illnesses), and physiologic information to calculate each patient's predicted risk of in-hospital death using the New Simplified Acute Physiology Score [7]. This score is a validated method of predicting in-hospital mortality for surgical and medical ICU patients [8,9].

### Identifying Adverse Clinical Occurrences

We used two steps to detect adverse events. First, we performed active surveillance for *adverse clinical occurrences*, which we defined as any incident in which there was an undesirable change in patient status. These included: the development of new significant laboratory findings (for example, a drop in haemoglobin by more than 20%); orders for medications that could indicate an AE (for example, naloxone); an important change in the patient's status (for example, the development of a new fever); the diagnosis of specific conditions (such as deep vein thrombosis, ventilator associated pneumonia, or central line associated blood stream infection); and the occurrence of specific system events (such as a perceived inability to obtain consultation in a timely manner, or an IV pump error). These criteria were modified from those used in a previous ICU-based patient safety study [10]. Additional file 1 lists all criteria used to define adverse clinical occurrences in the study.

An ICU nurse (not directly involved in patient care but working for the study alone) monitored patients for adverse clinical occurrences while they remained in the ICU. Surveillance occurred Mondays through Fridays from 7:30 am until 3:30 pm and consisted of several activities. First, the nurse attended morning rounds with the clinical team. Second, the nurse also remained in the ICU throughout the day. During these times, she interacted with all ICU staff, including nurses, physicians, pharmacists, respiratory therapists, and clerks. These interactions included questions pertaining to the practitioners' knowledge of any potential adverse clinical occurrences or the spontaneous volunteering of such information. Finally, the nurse reviewed all patient records for patients admitted to the ICU each day.

Adverse clinical occurrences that occurred in the ICU outside of the hours of direct observation were identified in several ways. Usually, they were discussed openly at rounds on the subsequent day. In addition, providers would often volunteer information. Finally, we relied to a certain extent upon documentation in the medical record. We did not monitor patients for adverse occurrences after they left the ICU.

The nurse recorded specific information about each adverse clinical occurrence. This included: a detailed description of the occurrence; all treatments the patient was receiving at the time of the occurrence; the health team's reaction to the occurrence; the patient's response to this reaction; and the documented cause of the occurrence. The nurse obtained this information by reviewing the medical record and interviewing relevant nurse(s), resident physician(s), and attending intensivist physician(s).

We used a peer review process to determine whether the adverse clinical occurrence was an *adverse event*. This process was used to discriminate between occurrences that were due to the patient's underlying condition versus those that were a result of medical care. We used an implicit review process as developed by Brennan and colleagues [11] and used by other investigators [2,3,12-15]. We adapted this method so that all AEs could be reviewed by a multidisciplinary panel, as was done in previous studies [10,16]. Each week during the study, a multi-disciplinary panel reviewed all adverse clinical occurrences from the preceding seven days. The panel consisted of one of the intensivist physicians involved in patient care for the ICU that week, an ICU nurse, the nurse observer, and a hospital-based internist (AJF). Note the intensivist was not the physician of record but was the back up physician for the week and the nurse was not directly involved in any patient care responsibilities. Thus, they were not conflicted in terms of their propensity to rate cases as errors. However, their direct knowledge of the cases allowed them to make more informed ratings. For each adverse clinical occurrence, the review panel determined whether the occurrence was truly an event in which the patient's status changed. If so, the panel rated whether treatment was the cause of the occurrence versus the underlying disease process. If the panel judged that the occurrence was due to treatment, the occurrence was classified as an *adverse event*. All AEs were reviewed by the panel to determine if they were avoidable with the available resources and currently accepted practices. If so, the event was classified as a *preventable adverse event*.

### Study outcomes

The primary outcome was *time to in-hospital death*. The secondary outcome was *time from ICU admission to hospital discharge*. These outcomes were measured by clinical chart review and were done independently of AE ascertainment.

### Statistical analysis

All analyses were conducted using SAS v9.1.3 (Cary, N.C.). We described patient characteristics and proportion of patients with AEs. For all proportions, we calculated 95% confidence limits using the Wilson Score method. The incidence AE rate was calculated as the number of AEs per ICU patient days. We compared characteristics of patients who did and did not experience an AE using the Wilcoxon Rank Sum Test for continuous variables and the chi-square statistic for categorical variables.

We determined whether AEs were associated with time to in-hospital death or time to hospital discharge using Cox proportional hazards modelling. In all models, AE status was expressed as a time-dependent covariate. We generated two models for each outcome. Model 1 included 'time to first AE' as its independent variable of interest. Model 2 included 'time to first preventable AE' and 'time to first non-preventable AE' as the independent variables of interest. To determine whether Model 2 was more informative than Model 1, we performed a likelihood ratio test. The result of this test was non-significant for both outcomes. For the time to death analyses, we censored patient observation at hospital discharge for patients discharged alive. For the time to hospital discharge analyses, we censored patient observation at death. All models included the following covariates: age; probability of death calculated using the New Simplified Acute Physiology Score; number of days in hospital prior to ICU admission; and Charlson score to quantify chronic comorbidities. The New Simplified Acute Physiology Score is a validated method for predicting the probability of in-hospital mortality. In our model, we estimate the change in risk associated with a 10% increase in the probability of death. We stratified each model according to the patient location immediately prior to ICU admission (i.e., emergency department, operating room, general ward).

The association between AE occurrence and the outcomes of interest were reported as hazard ratios (i.e., the ratio of risk for the outcome in patients with an AE vs. those without an AE). For the time to hospital discharge analysis, a hazard ratio less than one indicates that the probability of discharge at any time is less in patients with an AE.

To identify the impact of AEs on hospital length of stay, we generated and compared three survival curves. Using the Kaplan Meier method, we first plotted unadjusted survival curves of the proportion of patients remaining in hospital alive versus days in hospital for patients with and without adverse events. Then, using the BASELINE STATEMENT in PROC PHREG, we generated an adjusted survival function using our Cox model for adverse event patients under the condition that an adverse event *never occurred*. Finally, to calculate the impact of adverse events on length of stay, we compared the median length of stay for each of these curves.

## Results

Table 1 describes the 207 patients in the study. Patients were predominantly male, elderly, and most had been admitted from a surgical service primarily directly from the Emergency Department or the Operating Room. The patient population was very ill: 81% of patients required mechanical ventilation in the first 24 hours following ICU admission; 50% of patients had a Glasgow Coma Scale less than 8; and the median predicted mortality was 31%.

**Table 1:** Study patient characteristics

**Characteristics**	**N = 207**
Female	75 (36%)
Median Age (25th – 75th %ile)	66 (54 – 75)
Median Glasgow Coma Scale (25th – 75th %ile)	8 (6 – 11)
Median Probability of Death (25th – 75th %ile)*	31 (13 – 62)
Charlson Index	
0	62 (30%)
1	92 (44%)
2	39 (19%)
3	14 (7%)
Admitting Service	
Medicine	86 (42%)
Surgery	121 (58%)
Diabetes	
without end organ damage	19 (9%)
with end organ damage	9 (4%)
Indication for ICU admission	
Medical	131 (63%)
Post operative (scheduled)	33 (16%)
Post operative (unscheduled)	19 (9%)
Trauma	24 (12%)
Ventilation required in first ICU day	160 (81%)
Location prior to ICU admission	
ER	79 (38%)
OR	60 (29%)
Hospital floor	31 (15%)
Outside hospital	19 (9%)
Intermediate care unit	11 (5%)
Other	7 (3%)

*Probability of death calculated according to the New Simplified Acute Physiology Score [7].

Intermediate care = Medical and surgical step down units and the Post-anaesthetic care unit. Unless otherwise indicated, the number and percentage of patients with characteristic is presented.

Table 2 and Additional file 1 (Appendix 2) describe each AE. During a median ICU length of stay of 5 days (IQR 2–10 days), 56 AEs occurred in 40 patients (19% [95% CI 15%–25%]). The 56 AEs consisted of 7 different types with the most common being procedural complications (n = 18 (32%)), nosocomial infections (n = 13 (23%)), and adverse drug events (n = 12 (21%)). The median time from ICU admission to AE was 3.5 days (IQR = 1–8.5 days). The panel deemed 23 AEs occurring in 21 patients as preventable (10% [95% CI 7%–15%]). The 23 preventable AEs consisted of five different AE types with the most common being nosocomial infections (n = 8 (35%)), therapeutic errors (n = 5 (22%)) and procedural complications (n = 6 (26%)).

**Table 2:** Adverse event types

**Type**	**All AEs n (%)**	Preventable AEs, n (%)*	**Non-preventable AEs, n (%)**
Total	56 (100)	23 (100)	33 (100)
Procedural complication	18 (32)	6 (26)	12 (36)
Nosocomial infection	13 (23)	8 (35)	5 (15)
Adverse drug event	12 (21)	2 (9)	10 (30)
Surgical complication	6 (11)	0 (0)	6 (18)
Therapeutic error	5 (9)	5 (22)	0 (0)
System error	1 (2)	1 (4)	0 (0)
Diagnostic error	1 (2)	1 (4)	0 (0)

AE = adverse event. Non-preventable AEs were deemed unavoidable by the multidisciplinary review panel.

Fifty-two patients died during the hospitalization (25%, 95% CI = 20%–31%) with a median time from ICU admission to death of 9 days (IQR = 2.5–15 days). Thirty-six patients died while in the ICU, the remainder dying on a hospital ward after discharge from ICU. For patients discharged alive, the median length of hospital stay after ICU admission was 15 days (IQR = 8–34 days). Twelve patients were still alive in hospital at the time of study completion.

Patient characteristics were not associated with AE occurrence. Table 3 shows that none of the patient characteristics differed significantly between those with or without an AE. None of these patient characteristics were associated with time to AE in the survival model.

**Table 3:** Characteristics of patients by adverse event status

**Characteristics**	**Patients with AE**	**Patients without AE**	**P value**
N	40	167	
Female – no. (%)	17 (42.5)	58 (34.7)	0.36
age – median yr. (25th – 75th %ile)	65 (53–75)	66 (55–76)	0.61
Glasgow coma scale – median (25th – 75th %ile)	7 (5–10)	8 (6–11)	0.07
Probability of death – median % (25th – 75th %ile)*	0.34 (0.17–0.70)	0.31 (0.12–0.62	0.45
Charlson Index – no. (%)			0.49
0	11 (27.5)	51 (30.5)	
1	18 (45.0)	74 (44.3)	
2	10 (25.0)	29 (17.4)	
3	1 (2.5)	13 (7.8)	
Admitting Service – no. (%)			0.76
Medicine	15 (37.5)	71 (42.5)	
Surgery	25 (62.5)	96 (62.5)	
Diabetes – no. (%)			0.16
without end organ damage	1 (2.5)	18 (10.8)	
With end organ damage	3 (7.5)	6 (3.6)	
Indication for ICU admission – no. (%)			0.09
Medical reasons	21 (52.5)	110 (65.9)	
Post-op (scheduled)	5 (12.5)	28 (16.8)	
Trauma	7 (17.5)	17 (10.2)	
Post-op (unscheduled)	7 (17.5)	12 (7.2)	
Ventilation required in first 24 hrs – no. (%)	36 (90)	131 (78.4)	0.09
Location prior to ICU admission – no. (%)			0.70
Emergency department	14 (35)	65 (38.9)	
Operating room	11 (18.3)	49 (29.3)	
Hospital floor	7 (17.5)	24 (14.4)	
Outside hospital	3 (7.5)	16 (9.6)	
Other	3 (7.5)	4 (2.4)	
Intermediate care	2 (5.0)	9 (5.4)	

*Probability of death calculated according to the New Simplified Acute Physiology Score [7].

ICU = Intensive Care Unit; AE = Adverse event; Intermediate care = Medical and surgical step down units and the Post-anaesthetic care unit.

AEs were not associated with time to in-hospital death, either before or after adjusting for factors also associated with hospital mortality (Table 4a). The hazard ratio, which measures the daily risk of death for patients with vs. without AEs, was 0.93 (95% CI: 0.44–1.98). For preventable AEs, there was a trend towards decreased risk of death (HR = 0.72 (95%CI: 0.25–2.04)).

**Table 4:** Effect of adverse events (AE) on patient outcomes.

**a) Effect of AEs on time to death**	
**Variable**	**Hazard ratio (95% CI)**

**Model 1**	
AE	0.93 (0.44–1.98)
Age	1.0 (0.98–1.02)
Probability of death	1.41 (1.24–1.60)
LOS prior to ICU admit	0.94 (0.86–1.02)
Charlson score	1.13 (0.96–1.32)

Model 2*	
Preventable AE	0.72 (0.25–2.04)
Non-preventable AE	0.69 (0.27–1.76)

CI = Confidence interval.	
**b) Effect of AEs on time to discharge and length of stay**	

**Variable**	**Hazard ratio (95% CI)**
**Model 1:**	
AE	0.50 (0.31–0.81)
Age	1.00 (0.99–1.01)
Probability of death	0.84 (0.78–0.91)
LOS prior to ICU admit	0.99 (0.96–1.02)
Charlson score	0.88 (0.77–1.00)
Model 2*:	
Preventable AE	0.46 (0.23–0.91)
Non-preventable AE	0.54 (0.30–1.00)

CI = Confidence interval.	

These tables present the association of ICU-based adverse events (AEs) with time to death (section a) and time to hospital discharge (section b). For each outcome, two models are presented: in model 1 we modelled time to first AE as the independent variable of interest; in model 2 we modelled time to first preventable AE and non-preventable AEs as the independent variables of interest. In all models, adverse events were expressed as time-dependent factors. All models were stratified by patient location prior to ICU admission. For brevity, we omitted the hazard ratios for these covariates for Model 2 from the table as they do not differ from the estimates presented for Model 1. The hazard ratio for probability of death represents the increase in risk expected with a 10% increase in the new Simplified Acute Physiology Score [7].

AEs were strongly associated with time to hospital discharge, even after adjusting for other important clinical factors (Table 4b). The hazard ratio, which measures the daily risk of hospital discharge for patients with vs. without AEs, was 0.50 (95% CI: 0.31–0.81).

**Figure 1:**
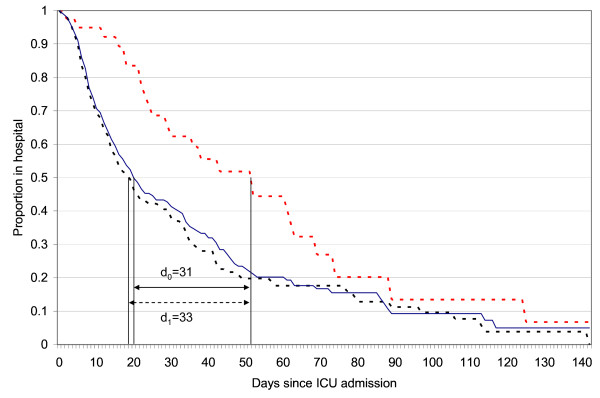
**Impact of ICU-based adverse events on hospital length of stay**. We have plotted three survival functions: an unadjusted survival function for patients with an adverse event (red dotted line); an unadjusted survival function for patients without an adverse event (black dotted line); and, the expected survival function for patients with an adverse event in the event they actually did not have an adverse event (blue solid line). The expected survival function was calculated with the values of model covariates for all cases. This Cox model included the following covariates: age, probability of death as measured by the new Simplified Acute Physiology Score, length of stay prior to ICU admission, and Charlson score. The median lengths of stay are indicated on the curve by black vertical lines. The differences in length of stay are presented.

Figure 1 illustrates the impact of AEs on length of stay. The unadjusted median lengths-of-stay for patients with and without AEs were 19 and 52 days, respectively. Our model based estimate of the median length of stay in AE patients under the condition that they did not have an AE was 21 days. Thus, experiencing an AE in the ICU appears to translate to an average increase in the length of hospital stay of 31 days.

The impact of AE status on hospital length of stay did not differ by preventability status. The hazard ratios for preventable and non-preventable AEs on hospital length of stay were essentially equivalent (HR = 0.46, 95% CI: 0.23–0.91 versus HR 0.54, 95% CI: 0.30–1.0, respectively).

## Discussion

Our study has three major findings. First, it confirms that ICU patients face a high risk of treatment related injury. Close to 20% of the patients in our study experienced an AE while in the ICU and one in five AEs was considered preventable. Second, we quantified the impact that ICU-based AEs have on patients and the health system. We estimated that AEs were independently associated with an average increase in hospital length of stay of 31 days. This association was similar in magnitude and significance for preventable and non-preventable AEs. We did not find a significant statistical association of AEs and mortality. Third, we described the types of AEs that affect ICU patients. In doing so, we found several classes of AEs and preventable AEs. Not one of these classes represented more than a third of all AEs.

This study reaffirms the importance of improving patient safety in the ICU by measuring the risk of AEs and more accurately quantifying their impact. Rothschild et. al. used a surveillance protocol similar to ours and found that 20% of ICU patients had an AE [10]. In this study, however, patient outcomes were known when the AEs clinical impact was rated. The investigators rated AEs in terms of their clinical impact: 'significant', 'severe', 'life-threatening', or 'fatal. While such a rating scheme is descriptive, it does not inform regarding downstream impact of the AEs. Furthermore, it does not help us to separate out the severity of the patient's underlying illness from the contribution by the AE. Finally, this methodology is potentially biased because the reviewer may be influenced by patient outcome [5,10]. These issues did not influence our study as much because the outcomes of interest were objective, precisely defined, and ascertained independent of the AE rating. (Note that only two patients died at the time of our rating. Otherwise all outcomes occurred after the review.) We believe that our study adds to the AE literature by providing a more valid and quantifiable estimate of AEs impact on patient outcomes.

Our results highlight the challenge faced by researchers trying to demonstrate measurable improvements in patient safety. Like Rothschild et. al. [10], we found several diverse types of preventable AEs. This diversity suggests that one single patient safety intervention is unlikely to effectively reduce all preventable AEs, especially since most quality improvement interventions have, at best, a modest impact on outcomes [17,18].

Our study is consistent with other studies showing that AEs predict an increased length of stay [19-23]. These prior studies differed from ours in several important ways: they evaluated patients not representative of adults in North American ICUs; they used different methodologies to capture information on complications; and, they did not perform survival analyses, which accounted for the time dependant nature of AEs. Nevertheless, the consistent findings strengthen our assertion that there is a need to find ways to reduce ICU-based AEs.

The methods we used were rigorous and defendable. We chose to study ICU patients, who have been previously demonstrated to be at high risk of AEs [10]. Therefore, studying them offered us a relatively efficient way to study this question. Our prospective design ensured that we could identify important covariates for all patients in an unbiased fashion and ensure a more accurate AE detection than previous studies. We excluded AEs occurring prior to ICU admission, as we did not have resources to perform surveillance on all hospitalized patients. However, we adjusted for time in hospital before ICU admission in our model. Our outcomes were objective and were ascertained independently of AE determination. Finally, we used appropriate analytic methods to account for important confounding variables and the time-dependent nature of AEs [24].

Our study has several limitations. First, we studied ICU patients only. Due to the critical condition of these patients and their aggressive monitoring and treatment, one might expect the effects of AEs to be greater in this population. Thus, we might find a diminished association between AEs and outcomes if we studied patients treated in other areas of the hospital. Second, our secondary outcome, length of stay, is not entirely patient-centered. Other outcomes such as pain, anxiety or functional status, which may be more sensitive to the effect of AEs and preventable AEs, might be more relevant to patients. We chose not to study these outcomes as they are more difficult to reliably measure in an objective manner, especially in critically ill patients. Furthermore, an increase in length of stay is important from a patient perspective as it is often necessitated for treatment of debilitating and painful conditions. Third, as previously mentioned, we had a relatively small sample size, increasing the possibility that we failed to detect true associations between preventable AEs and important outcomes. Fourth, some of the reviewers were participating in some patient care responsibilities indirectly, as they were considered back-up providers. Although this might lead to a propensity to avoid rating cases of errors, we do not feel this occurred. Furthermore, this potential bias would not influence the measured association of the AEs with length of stay and mortality.

## Conclusion

In conclusion, ICU-based AEs are common and have a large impact on hospital length of stay. Reducing their impact will be challenging because preventable AEs are less common and have many different causes. These data suggest that efforts to improve outcomes for ICU patients might be more effective if they focus on new technologies and treatment methods rather than focusing on error reducing strategies exclusively.

## Competing interests

The authors declare that they have no competing interests.

## Authors' contributions

AJF made substantial contributions to conception and design of the study. He participated in the acquisition of data. He led the analysis and interpreted the data. AJF drafted the manuscript. AJF gave final approval for the version to be published. KK participated in the acquisition of data. He participated in the interpretation of the data. He revised the manuscript for important intellectual content. KK gave final approval for the version to be published. JH participated in the acquisition of data. He participated in the interpretation of the data. He revised the manuscript for important intellectual content. JH gave final approval for the version to be published. KS made substantial contributions to conception and design of the study. He participated in the interpretation of the data. He revised the manuscript for important intellectual content. KS gave final approval for the version to be published. CvW made substantial contributions to conception and design of the study. He analyzed the data. He revised the manuscript for important intellectual content. CvW gave final approval for the version to be published.

## Pre-publication history

The pre-publication history for this paper can be accessed here:



## Supplementary Material

Additional file 1 **Appendix 1 and 2**. Appendix 1 Criteria used for defining adverse clinical occurrences. Appendix 2 Adverse Events. Click here for file
