# Supplementary material for: The impact of adverse events in the intensive care unit on hospital mortality and length of stay
Source: BMC Health Serv Res. 2008 Dec 17;8:259. doi: 10.1186/1472-6963-8-259 (PMC2621200; doi:10.1186/1472-6963-8-259)
Supplement: Additional file 1 — Appendix 1 and 2. Appendix 1 Criteria used for defining adverse clinical occurrences. Appendix 2 Adverse Events. [file 1472-6963-8-259-S1.doc]

**Appendix 1 Criteria used for defining adverse clinical occurrences**

| **Laboratory based triggers** | **Diagnosis based triggers** |
| --- | --- |
| INR >5 | New DVT/PE |
| K<2.8, K>6.0 | New Pressure Ulcer |
| Na <120, Na >150 | Fall resulting in injury |
| Cr >25% change | Altered Level of consciousness |
| AST>500 or 3-fold increase | Family/Patient dissatisfied with care |
| ALT>500 or 3-fold increase | Ventilator associated pneumonia |
| tBili>30 or 3-fold increase | CV Line infection |
| Alp >250 | CV Line complication – pneumothorax |
| CK>500 | CV Line complication - arterial injury |
| TNT>0.10 | CV Line complication - difficult insertion (>3 attempts) |
| Glucoscan<3.0 | CV Line complication – other |
| Glucoscan>18 *2 | Arterial Line complication |
| WBC<3.0 | Inadequate sedation |
| WBC>18 | Excessive sedation |
| Hb decrease > 20% | Failure to wean from ventilation |
| Plt decrease by > 50% | Ogilvies syndrome |
| Calcium<1.5 | Pneumothorax |
| Calcium>3.0 | Return to OR |
| Phospate<0.4 | New gastric/duodenal ulcer |
| Mg <0.4 | ETT complications: extubation |
| Mg > 5.0 | ETT complications: hypotension |
| pH<7.25 | ETT complications: trauma |
| pH>7.5 | ETT complications: aspiration |
| HIT assay positive | ETT complications: prolonged hypoxemia |
| Positive blood culture | **System based triggers** |
| Positive c.difficile toxin assay | Stat ECG |
| **Pharmacy based triggers** | New Isolation Precautions |
| Order for flumazenil | Use of physical restraints |
| Order for nalaxone | Admission due to rapid response team call |
| Order for Vitamin K | Admission due to cardiac arrest |
| Order for Protamine | Unable to obtain MD in a timely manner |
| Order for FFP | Unable to obtain consultant in a timely manner |
| **Patient based triggers** | Medication administration error |
| Respiratory distress/Dyspnea | Equipment unavailable in emergency situation |
| Chest pain | Medical equipment problem |
| O2 sat <90% | Nightime/Weekend admission |
| Systolic BP <100 | IV pump error |
| HR>110 | Delay in therapy |
| Temp>39C, Temp<35C | OR cancellation |
| Rash | Diagnostic Error |
| Nausea/Vomitting | **Other** |
| Unexpected death |  |

**Appendix 2 Adverse Events**

| **ID** | **Description** | **Preventable** | **Class** |
| --- | --- | --- | --- |
| 1 | Bleeding in a post operative patient put on anti-coagulants for questionable indication. | YES | Therapeutic error |
| 2 | Pulmonary edema causing respiratory failure as a result of a delay in instituting hemodialysis. | YES | Therapeutic error |
| 3 | Several errors in diagnosis and management leading to the death of a critically ill patient. | YES | Diagnostic error |
| 4 | Dislocated jaw secondary to intubation. There was a several week delay in making the diagnosis. | YES | Procedural complication |
| 5 | Pt develops abdominal hematoma secondary to IV heparin. There was a delay in diagnosis. | YES | ADE |
| 6 | Patient developes VAP following delayed surgery. Patient was ventilated unnecessarily while awaiting operation. | YES | System error |
| 7 | Inadvertent damage to bowel during the emergency repair of a ruptured AAA. | NO | Surgical complication |
| 8 | Intracerebral bleed complicating thrombolytic treatment. | NO | ADE |
| 9 | Post operative MI in AAA repair patient | NO | Procedural complication |
| 10 | Surgical infection | NO | Surgical complication |
| 11 | Post-operative bleeding | NO | Surgical complication |
| 12 | Surgical infection | NO | Surgical complication |
| 13 | VAP | YES | Nosocomial infection |
| 14 | VAP | YES | Nosocomial infection |
| 15 | VAP | YES | Nosocomial infection |
| 16 | CVC-associated infection | NO | Procedural complication |
| 17 | Vancomycin associated acute renal failure | NO | ADE |
| 18 | VAP | YES | Nosocomial infection |
| 19 | CVC-associated infection | YES | Procedural complication |
| 20 | Post operative bleeding and ischemic bowel. | NO | Surgical complication |
| 21 | VAP | YES | Nosocomial infection |
| 22 | VAP | YES | Nosocomial infection |
| 23 | VAP | YES | Nosocomial infection |
| 24 | VAP | YES | Nosocomial infection |
| 25 | Surgical infection | NO | Surgical complication |
| 26 | Difficulty with line insertion resulting in neck hematoma. | YES | Procedural complication |
| 27 | Patient fall | YES | Therapeutic error |
| 28 | Hypoglycemia in patient on insulin drip. Protocol for monitoring glucose was not followed. | YES | ADE |
| 29 | Arterial injury during CVC insertion | YES | Procedural complication |
| 30 | Arterial injury during CVC insertion | YES | Procedural complication |
| 31 | Bleeding on heparin. Heparin was not indicated. | YES | Therapeutic error |
| 32 | Delayed response from consultant leading to prolonged patient distress. | YES | Therapeutic error |
| 33 | CVC associated bleeding | NO | Procedural complication |
| 34 | Pressure ulcers | NO | Procedural complication |
| 35 | Pneumothorax following chest tube insertion. | NO | Procedural complication |
| 36 | Pneumothorax following thoracentesis. | NO | Procedural complication |
| 37 | Heparin induced thrombocytopenia | NO | ADE |
| 38 | Hypotension following intubation | NO | Procedural complication |
| 39 | Clostridium difficile infection | NO | Nosocomial infection |
| 40 | Bleeding secondary to concomitant CVC and anticoagulants | NO | ADE |
| 41 | Medication rash | NO | ADE |
| 42 | Dilantin induced leucopenia | NO | ADE |
| 43 | Central nervous system MRSA infection | NO | Nosocomial infection |
| 44 | Pneumothorax following chest tube insertion. | NO | Procedural complication |
| 45 | Failed extubation | NO | Procedural complication |
| 46 | Excessive sedation | NO | ADE |
| 47 | Medication rash | NO | ADE |
| 48 | Delirium | NO | Procedural complication |
| 49 | Heparin induced thrombocytopenia | NO | ADE |
| 50 | Transfusion reaction | NO | ADE |
| 51 | Medication rash | NO | ADE |
| 52 | CVC associated DVT | NO | Procedural complication |
| 53 | Pressure ulcer | NO | Procedural complication |
| 54 | Medication rash | NO | ADE |
| 55 | Bacteremia | NO | Nosocomial infection |
| 56 | CVC associated bleeding | NO | Procedural complication |

VAP=ventilator associated pneumonia; AAA=abdominal aortic aneurysm; CVC=central venous catheter; MRSA=methicillin resistant staphylococcus aureus; DVT = deep venous thrombosis
